# 

**Published:** 1857-11

**Authors:** 


*Apologetical.*—We owe our indulgent readers an apology for seeming negligence in the issue of the Journal for the past two months. The State Fair unfortunately happened at the time when our October number should have been issued, and though every effort on our part was used, it was impossible to be in with the first of of the month ; thinking to appease our subscribers by an exceedingly prompt issue in November, we prepared the copy very far in advance. Another and more serious source of difficulty was a general strike of the printers throughout the city, leaving upon the ground only one faithful and most competent hand, who has "set" on it like a persistent and motherly hen for the last dozen years. We shall be all right hereafter, in fact are already so, so far as our publishing arrangements are concerned. F.

---

*Editorial Changes.*—We notice the name of Prof. Fenner on the cover of the New Orleans Medical News and Hospital Gazette, as assistant editor; Drs. Choppin and Beard having retired. In his salutatory, Dr. Fenner draws rather a discouraging picture of medical editorship, as far as remuneration is concerned. After four years assiduous labor in support of the New Orleans Medical and Surgical Journal, he received the amount of *one hundred and twenty-five dollars.* In spite of this discouraging fact, Dr. Fenner has again donned the "editorial harness," and we cordially welcome him back to a field from which he has reaped fame if not dollars and cents.

F.

---

*To our Subscribers.*—We are compelled to close this number with a dun, but of no ordinary character. We are fully sensible of the hard times and the difficulty of paying anything, but we must ask every delinquent subscriber for the small sum of our subscription; we would not ask for two hundred dollars, for we would be certain not to get it, but the small sums which are due to us from so many, could certainly be paid without great inconvenience. Send us money of any description which is current where it comes from, at our risk.

